# Test-retest reliability and four-week changes in cardiopulmonary fitness in stroke patients: evaluation using a robotics-assisted tilt table

**DOI:** 10.1186/s12883-016-0686-0

**Published:** 2016-09-06

**Authors:** Jittima Saengsuwan, Lucia Berger, Corina Schuster-Amft, Tobias Nef, Kenneth J. Hunt

**Affiliations:** 1 Institute for Rehabilitation and Performance Technology, Division of Mechanical Engineering, Department of Engineering and Information Technology, Bern University of Applied Sciences, Burgdorf, Switzerland; 2 ARTORG Center for Biomedical Engineering Research, Gerontechnology and Rehabilitation Research Group, University of Bern, Bern, Switzerland; 3 Research Department, Reha Rheinfelden, Rheinfelden, Switzerland; 4 Department of Physical Medicine and Rehabilitation, Faculty of Medicine, Khon Kaen University, Khon Kaen, Thailand

**Keywords:** Cardiopulmonary exercise testing, Robotics-assisted tilt table, Stroke, Test-retest reliability, Repeatability

## Abstract

**Background:**

Exercise testing devices for evaluating cardiopulmonary fitness in patients with severe disability after stroke are lacking, but we have adapted a robotics-assisted tilt table (RATT) for cardiopulmonary exercise testing (CPET). Using the RATT in a sample of patients after stroke, this study aimed to investigate test-retest reliability and repeatability of CPET and to prospectively investigate changes in cardiopulmonary outcomes over a period of four weeks.

**Methods:**

Stroke patients with all degrees of disability underwent 3 separate CPET sessions: 2 tests at baseline (TB1 and TB2) and 1 test at follow up (TF). TB1 and TB2 were at least 24 h apart. TB2 and TF were 4 weeks apart. A RATT equipped with force sensors in the thigh cuffs, a work rate estimation algorithm and a real-time visual feedback system was used to guide the patients’ exercise work rate during CPET. Test-retest reliability and repeatability of CPET variables were analysed using paired t-tests, the intraclass correlation coefficient (ICC), the coefficient of variation (CoV), and Bland and Altman limits of agreement. Changes in cardiopulmonary fitness during four weeks were analysed using paired t-tests.

**Results:**

Seventeen sub-acute and chronic stroke patients (age 62.7 ± 10.4 years [mean ± SD]; 8 females) completed the test sessions. The median time post stroke was 350 days. There were 4 severely disabled, 1 moderately disabled and 12 mildly disabled patients. For test-retest, there were no statistically significant differences between TB1 and TB2 for most CPET variables. Peak oxygen uptake, peak heart rate, peak work rate and oxygen uptake at the ventilatory anaerobic threshold (VAT) and respiratory compensation point (RCP) showed good to excellent test-retest reliability (ICC 0.65–0.94). For all CPET variables, CoV was 4.1–14.5 %. The mean difference was close to zero in most of the CPET variables. There were no significant changes in most cardiopulmonary performance parameters during the 4-week period (TB2 vs TF).

**Conclusions:**

These findings provide the first evidence of test-retest reliability and repeatability of the principal CPET variables using the novel RATT system and testing methodology, and high success rates in identification of VAT and RCP: good to excellent test-retest reliability and repeatability were found for all submaximal and maximal CPET variables. Reliability and repeatability of the main CPET parameters in stroke patients on the RATT were comparable to previous findings in stroke patients using standard exercise testing devices. The RATT has potential to be used as an alternative exercise testing device in patients who have limitations for use of standard exercise testing devices.

## Background

Stroke is one of the leading causes of adult disability worldwide [1, 2]. Stroke affects not only the neurological system but also influences pulmonary and cardiovascular health [3, 4]. The effect of stroke on the cardiovascular system can result from: existing comorbid conditions, e.g. coronary artery disease; a direct effect of stroke on impairments in cardiovascular regulation, e.g. stroke patients frequently have electrocardiographic abnormalities such as T-wave abnormalities, prolonged QTc interval and arrhythmia [5]; or, indirectly from weakness, fatigue or spasticity leading to a sedentary lifestyle and resulting in a further decline in cardiopulmonary fitness [6].

Cardiopulmonary exercise testing (CPET) is important to determine a patient’s cardiopulmonary fitness and to accurately prescribe an individualised exercise programme [7]. The most commonly used devices, i.e. a treadmill or a cycle ergometer, cannot be used in all stroke patients – their use is limited to mildly or moderately impaired stroke patients [8]. Post stroke impairments such as spasticity, muscle weakness, coordination problem or reduced joint range of motion may preclude some moderately to severely impaired stroke patients from performing CPET on these devices [9, 10]. Recent systematic reviews showed that suitable methods to measure cardiopulmonary fitness and to provide appropriate exercise training in stroke patients with severe disability are still lacking [11, 12]. This problem needs to be addressed soon because stroke incidence is projected to increase as a result of a higher proportion of older people in the population in the future [13] and because people who live with disability after stroke have a longer survival than formerly [14].

In this study, a robotics-assisted tilt table (RATT), which is used clinically for early neurorehabilitation, was adapted for CPET. This device was considered to have potential to be used in severely disabled patients because it has a harness to provide body support and it incorporates thigh cuffs and foot plates to secure the legs [15]. As the patient is well supported, the risk of falls or exercise related injuries is minimised. The RATT was augmented with force sensors, a work rate calculation algorithm and a visual feedback system to facilitate exercise testing and training [16]. A previous validity and reliability study in healthy individuals found that peak oxygen uptake and submaximal exercise thresholds on the RATT were approximately 20 % lower than for a cycle ergometer [17]. Test-retest reliability of maximal and submaximal exercise thresholds on the RATT were comparable to standard exercise devices [17, 18]. Furthermore, a feasibility study in stroke showed that patients with severe disability could successfully undergo CPET and exercise training on the RATT [15].

Test-retest reliability of peak and submaximal cardiopulmonary exercise parameters, which are normally used as the main outcomes for exercise intervention studies, require careful analysis prior to future clinical uptake of the RATT. Although reliability data in healthy individuals have been established, this form of analysis in stroke patients with various degrees of disability is still lacking.

The aims of this study were twofold: to investigate test-retest reliability and repeatability of CPET on the RATT in stroke patients with all degrees of disability; and to prospectively investigate changes in cardiopulmonary outcomes in this sample over a period of four weeks.

## Methods

### Study design and participants

This prospective study was approved by the Ethics Review Committee of Canton Aargau, Switzerland (Ref. EKNZ 2014–296). All subjects gave their written informed consent before participating in the study.

Sub-acute and chronic stroke patients were recruited from Reha Rheinfelden, a rehabilitation centre in the north-west of Switzerland, from December 2014 to January 2016. Patient inclusion criteria were: (1) a diagnosis of first-ever stroke, either ischaemic or intracerebral haemorrhagic; (2) ≥ 18 years old; and (3) willing to cooperate in the study and able to attend all testing sessions. Exclusion criteria were: (1) any contraindications to maximal exercise testing according to the American College of Sports Medicine guidelines [19]; (2) any contraindications for the RATT based on guidelines from the manufacturer, e.g. body mass greater than 135 kg, bone instability, or open skin lesions in the area of the lower limbs and/or back; (3) severe perceptual or communication problems; (4) Mini Mental State Examination (MMSE) score ≤ 17 [20]; (5) concurrent neurological diseases, e.g. Parkinson’s disease; and (6) severe concurrent cardiac or pulmonary disease. A cardiologist evaluated the cardiac status of all patients before giving approval for participation.

### Study protocol

Patients underwent 3 separate CPET sessions: 2 tests at baseline (TB1 and TB2) and 1 test at follow up (TF). TB2 was conducted as soon as possible after TB1, but at least 24 h later. TB2 and TF were 4 weeks apart. Patients were instructed to avoid strenuous activity within the 24 h before each test session, not to consume a large meal 3 h before, and to refrain from caffeine and nicotine 12 h prior to testing.

### Patients’ characteristics

Gender, age, height, body mass, body mass index (BMI), type of stroke, time of stroke diagnosis, side of weakness, comorbidity and current medication were recorded prior to the first test (Table 1).

**Table 1:** Characteristics and demographic data of subjects

Characteristic (*n* = 17)	Value	Range
Age (years)	62.7 ± 10.4	41.0–78.0
Sex, n (%)		
Male	9 (52.9 %)	
Female	8 (47.1 %)	
Height (cm)	169.5 ± 6.6	160.0–183.0
Body mass (kg)	72.3 ± 10.0	57.5–90.0
Body mass index (kg/m^2^)	25.2 ± 3.2	20.2–33.1
Type of stroke, n (%)		
Ischaemic	15 (88.2 %)	
Haemorrhagic	2 (11.8 %)	
Hemiparetic side, n (%)		
Left	7 (41.2 %)	
Right	10 (58.8 %)	
Stage, n (%)		
Sub-acute	6 (35.3 %)	
Chronic	11 (64.7 %)	
Days post stroke,	485.3 (535.5)	21–1810
median (IQR)	350 (788)	
FAC	3.6 ± 2.1	0–5
NIHSS	3.1 ± 3.2	0–11
MMSE score	28.1 ± 2.3	21–30
Modified Rankin Scale		
0–2	12 (70.6 %)	
3	1 (5.9 %)	
4–5	4 (23.5 %)	
Comorbidities, n (%)		
Hypertension	9 (52.9 %)	
Diabetes mellitus	1 (5.9 %)	
Dyslipidemia	4 (23.5 %)	
None	4 (23.5 %)	
Antihypertensive medications, n (%)		
β-blocker	2 (11.9 %)	
ACE inhibitors	3 (17.6 %)	
Calcium channel blockers	3 (17.6 %)	
None	9 (52.9 %)	

Values are mean ± SD unless otherwise indicated

*Abbreviations*: *n* number; *SD* standard deviation; *MMSE* Mini Mental State Examination; *IQR* interquartile range; *FAC* functional ambulation category; *ACE* angiotensin-converting-enzyme

Prior to cardiopulmonary exercise testing, functional measures were assessed using functional ambulation category (FAC) [21], the National Institutes of Health Stroke Scale (NIHSS) [22] and a 6-min walk test (6 MWT) [23]. The 6 MWT was performed indoors along a straight 50 m walking course. Standardized instructions were given to the patients according to the guidelines. The six-minute walk distance (6MWD) was recorded.

### Cardiopulmonary fitness assessment

**Fig. 1:**
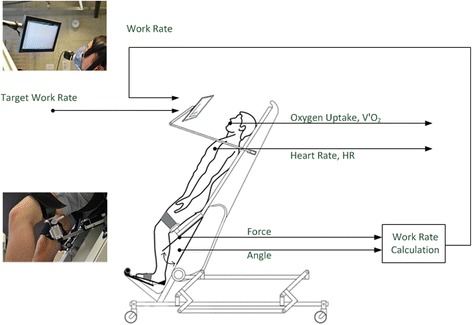
Robotics-assisted tilt table (RATT) with visual feedback system. The visual feedback screen shows the target work rate and the subject’s work rate. The latter was calculated from the forces in the thigh cuffs and the angular velocities. Adapted from Saengsuwan et al., 2015 [18]

Cardiopulmonary exercise testing (CPET) was performed using a robotics-assisted tilt table (RATT; model Erigo, Hocoma AG, Switzerland; Fig. 1). Each test session had the same format. The patient was first transferred and secured using the body harness, thigh cuffs and foot plates according to the instruction of the manufacturer. Then, the patient was tilted upwards to 70 ° and the formal CPET protocol described below was initiated. During the passive and incremental exercise phases, the stepping cadence was set at 80 steps/min, which is the maximal cadence allowed by the device.

The CPET methodology was previously described in detail elsewhere [15] and it is briefly summarized in the following. CPET consisted of the following phases: (1) a 3-min rest phase, where the patient lay without leg movement on the RATT; (2) a passive phase, where the RATT moved the patient’s legs for 5 min and the measured relative work rate was adjusted to 0 W; (3) a ramp phase, where the rate of work rate increase was in the range 1.25 to 4.5 W/min – the work rate ramp was estimated by patients’ gender, age, level of weakness and comorbidity, e.g. the ramp rate for a 70 year old woman with severe disability was set to 1.25 W/min – the aim of the individualised ramp rate was to bring the patient to their limit of functional capacity within 8–12 min of ramp onset. The individual ramp increment rates stayed the same for 13 patients in TB1 and TB2. The ramp rates were adjusted in four patients in TB2 to enable the patients to reach their maximal effort in 8–12 min; and (4) a recovery phase, where the patient remained passive while the RATT moved their legs for 5 min. The termination criteria for the ramp phase followed the American College of Sports Medicine guidelines [19]. Additionally, blood pressure (BP) was used as a termination criterion: systolic BP > 210 mmHg or diastolic BP > 115 mmHg [24].

Prior to the incremental exercise testing, the researcher showed and explained the Borg CR 10 scale to the patients. The instructions were as follows: “This scale aims to measure your feeling of difficulty in breathing and leg fatigue. The range of the scale is from 0 to 10. The number 0 means that you feel no difficulty at all. The scale progresses to number 10 where you feel that you have maximal difficulty with breathing/leg effort.” During the incremental exercise test the patients rated the Borg CR 10 for dyspnea every 3 min. At the end of exercise, the patients were asked to rate the Borg CR 10 for both dyspnea and leg effort.

Metabolic gas exchange was recorded throughout using a breath-by-breath system (MetaMax 3B, Cortex Biophysik GmbH, Germany); the data were analysed using the associated Metasoft software. Prior to each test, standardized pressure, volume and precision gas calibration were performed according to the manufacturer’s instructions. Heart rate was continuously recorded using a chest strap (model T34, Polar Electro Oy, Finland). Blood pressure was measured by manual sphygmomanometry every 2 min during the tests.

### Outcome measures

The primary outcome measures were:

1. Maximal outcomes:
  Peak oxygen uptake, denoted V'O_2peak_. This was determined as the maximum of a 15-breath average during the ramp phase.
  Peak heart rate, HR_peak_: the highest value of HR during the ramp phase.
  Peak respiratory exchange ratio, RER_peak_: the 15-breath average at the time of V'O_2peak_.
  Peak work rate, WR_peak_. This was calculated as the highest work rate achieved.
2. Submaximal outcomes:
  Oxygen uptake at the ventilatory anaerobic threshold VAT (V'O_2VAT_): the method for determination of VAT is summarized below.
  Oxygen uptake at the respiratory compensation point RCP (V'O_2RCP_), described below.

The secondary outcome measures were:

- Rating of perceived exertion (RPE) for dyspnea and leg effort at the time of peak exercise (Borg CR10) [25].
- Heart rate at the VAT (HR_VAT_) and heart rate at the RCP (HR_RCP_).
- The value of the ventilatory equivalent of oxygen (V'E/V'O_2_) at VAT and ventilatory equivalent of carbon dioxide (V'E/V'CO_2_) at RCP, where V'E is minute ventilation and V'CO_2_ is carbon dioxide output, and the slope of V'E-vs-V'CO_2_ from the start of the ramp phase to the RCP.

The ventilatory anaerobic threshold (VAT) and the respiratory compensation point (RCP) were determined as the averages from two independent raters (JS and LB) provided that the difference in the V'O_2_ values of the corresponding points between two raters was less than 100 mL/min [26]. In the case of any discrepancy, a third experienced rater (KH) rated the point, and the VAT or RCP was taken as the average of the 2 closest values. The methods used to determine the VAT and RCP were those described by Binder et al. [27] and summarised in the following.

The VAT was determined using the combination of these criteria: (1) the point where the V'E/V'O_2_ reaches its minimum or starts to increase without an increase in the V'E/V'CO_2_; (2) the point at which the partial pressure of end-tidal oxygen tension (P_ET_O_2_) reaches a minimum or starts to increase without a decline in the partial pressure of end-tidal carbon dioxide tension (P_ET_CO_2_); and, (3) the point of deflection of V'CO_2_versus V'O_2_ (V-slope method). The first two criteria were prioritized in the case that the 3 criteria gave different results.

The RCP was determined by: (1) the minimal value or nonlinear rise of V'E/V'CO_2_; (2) the point that P_ET_CO_2_ starts to decline; and, (3) the point of deflection of V'E versus V'CO_2_. Again, the first two criteria were prioritized.

### Statistical analysis

Continuous variables are presented as mean ± standard deviation (SD). Categorical variables are presented as frequencies and percentages. Test-retest reliability of submaximal parameters on each device was analysed using an intraclass correlation coefficient (ICC_3,1_) [28]. For the interpretation of results, 0.40 ≤ ICC < 0.75 was considered as fair to good reliability and ICC ≥ 0.75 was considered excellent reliability [29]. The within-subject coefficients of variation (CoV) were also calculated [30].

Repeatability was analysed using the Bland and Altman limits of agreement (LoA). Heteroscedasticity was examined by calculating Pearson’s correlation coefficient (r) between the absolute difference and the corresponding means. When *r* > 0.1 was found, the data were considered heteroscedastic. The heteroscedastic data were log transformed using base 10 and r was recalculated. If r decreased, the data were analysed using log-transformation. The limits of agreement for heteroscedastic data were transformed back and displayed in the Bland Altman plots as a linear function ± bx̄ calculated by the method described by Euser et al. [31], where x̄ is the data mean and b is the slope of the LoA. If the data were homoscedastic, or the data were heteroscedastic but the log-transformed data did not reduce the correlation coefficient, the limits of agreement were reported as the standard mean difference (MD) ± 1.96 SD of the difference [32].

Two-sided paired t-tests were used to test differences between TB1 and TB2, as well as between TB2 and TF, if the difference between the tests was normally distributed (Shapiro Wilk test). Otherwise, the Wilcoxon-signed rank test was used. The significance level was set at 0.05. The analyses were performed using SPSS (Version 20.0, IBM Corp., Armonk, NY).

## Results

### General observations

Seventeen patients (8 females, 9 males) aged 62.7 ± 10.4 years (mean ± SD), see Table 1, completed all three sessions and are included in the data analysis. The median time post stroke was 350 days. There were 4 severely disabled, 1 moderately disabled and 12 mildly disabled patients. Three further patients were recruited but did not complete all measurement sessions because of, respectively, severe hypertension, new onset atrial fibrillation, or due to automatic shutdown of the RATT caused by inappropriate forces. These three patients were not included in the data analysis

There were no complications or serious adverse events. The ramp-phase duration was 8 min 53 s ± 2 min 17 s. Of the total of 51 completed exercise test sessions (17 patients x 3 sessions each), 49 sessions were terminated at the patient’s own volition (functional capacity reached). The most common reasons for volitional exercise termination were leg fatigue (45.1 %), generalized fatigue (17.6 %) and inability to maintain the target work rate (15.7 %). One session was terminated because blood pressure reached the upper limit (SBP > 210 mmHg) and 1 session because of pain due to a tension headache.

**Fig. 2:**
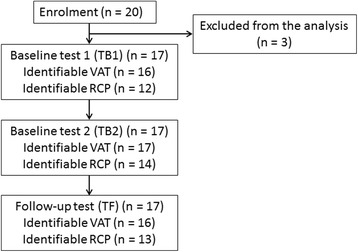
Study flow chart. Reasons for the three exclusions are detailed in results

Overall, the VAT and RCP were identifiable in 49 of 51 tests (96.1 %) and 39 of 51 tests (76.5 %), respectively: this allowed 16 paired comparisons of V'O_2VAT_ to be done for TB1 vs TB2 (test-retest) and for TB2 vs TF (four-week changes); 12 paired comparisons of V'O_2RCP_ were able to be done for TB1 vs TB2 and 11 comparisons for TB2 vs TF (Fig. 2, Tables 2 and 3). There was one measurement problem for submaximal heart rate recording in TB1, so the pairwise comparison for HR_VAT_ and HR_RCP_ were 1 pair lower than for the submaximal V'O_2_ pairs (test-retest).

**Table 2:** Test-retest reliability of the cardiopulmonary performance parameters

	TB1 (mean ± SD)	TB2 (mean ± SD)	p-value	MD	95%CI	SDD	95 % LoA	CoV (%)	ICC (95 % CI)
Absolute V'O_2peak_ (mL/min)	1130.5 ± 349.4	1156.4 ± 376.6	0.60	25.8	−77.4, 129.1	200.8	−0.26x̄, 0.26x̄	11.6	0.85 (0.64, 0.94)
Relative V'O_2peak_ (mL/kg/min)	15.7 ± 4.7	16.0 ± 4.9	0.65	0.3	−1.1, 1.7	2.8	−0.26x̄, 0.26x̄	11.6	0.84 (0.61, 0.94)
HR_peak_ (beats/min)	120.9 ± 26.3	124.2 ± 25.2	0.26	3.2	−2.6, 9.0	11.3	−18.9, 25.3	6.9	0.90 (0.76, 0.96)
HR_peak_% of APMHR	78.0 ± 15.0	80.1 ± 13.6	0.26	2.04	−1.6, 5.7	7.2	−12.1, 16.2	6.9	0.87 (0.69, 0.95)
RER_peak_	1.06 ± 0.09	1.08 ± 0.08	0.10	0.02	−0.01, 0.52	0.06	−0.1, 0.1	4.1	0.78 (0.49, 0.91)
WR_peak_ (W)	28.0 ± 12.4	29.0 ± 13.3	0.36	1.07	1.4, 3.5	4.6	−7.9, 10.1	13.4	0.94 (0.83, 0.98)
Absolute V'O_2VAT_ (mL/min) (*n* = 16)	575.4 ± 105.9	635.5 ± 177.3	0.045	60.1	−1.4, 118.9	110.2	−0.34x̄, 0.34x̄	14.5	0.67 (0.27, 0.87)
Relative V'O_2VAT_ (mL/kg/min)	8.0 ± 1.6	8.8 ± 2.4	0.050	0.8	0.0, 1.6	1.5	−0.34x̄, 0.34x̄	14.5	0.68 (0.29, 0.88)
V'O_2VAT_% of V'O_2peak_ (%)	50.3 ± 9.4	53.1 ± 9.1	0.27	2.8	−2.4, 8.2	10	−16.8, 22.4	15.4	0.41 (−0.07, 0.74)
HR_VAT_ (beats/min) (*n* = 15)	89.5 ± 9.3	91.9 ± 9.7	0.17	2.4	−1.1, 6.0	6.5	−10.3, 15.1	5.5	0.76 (0.44, 0.91)
HR_VAT_ % of HR reserve	32.6 ± 9.2	35.1 ± 11.5	0.33	2.5	−2.8, 7.8	9.5	−16.1, 21.1	19.5	0.58 (0.13, 0.84)
HR_VAT_ % of APMHR	57.3 ± 5.4	58.8 ± 5.2	0.20	1.5	−0.9, 3.8	4.3	−0.62x̄, 0.62x̄	5.5	0.67 (0.28, 0.87)
Absolute V'O_2RCP_ (mL/min) (*n* = 12)	956.5 ± 197.9	1002.9 ± 179.4	0.33	46.3	−54.1, 146.6	157.9	−263.2, 355.8	11.6	0.65 (0.17, 0.88)
Relative V'O_2RCP_ (mL/kg/min)	13.4 ± 2.5	14.1 ± 2.5	0.28	0.7	−0.7, 2.1	2.1	−3.4, 4.8	11.6	0.77 (0.24, 0.87)
V'O_2RCP_% of V'O_2peak_ (%)	77.2 ± 8.8	82.1 ± 6.8	0.035	4.9	0.4, 9.5	7.1	−9.0, 18.8	8.3	0.50 (−0.02, 0.82)
HR_RCP_ (beats/min) (*n* = 11)	112.5 ± 18.2	113.6 ± 17.7	0.42	1.1	−5.3, 7.4	9.4	−17.3, 19.5	5.8	0.87 (0.59, 0.96)
HR_RCP_ % of HR reserve	70.2 ± 16.2	73.6 ± 10.8	0.48	3.5	−7.0, 14.0	15.6	−27.1, 34.1	18.4	0.37 (−0.28, 0.78)
HR_RCP_ % of APMHR	71.3 ± 12.1	71.9 ± 11.7	0.72	0.7	−3.2, 4.6	5.8	−10.7, 12.1	5.8	0.89 (0.64, 0.97)
RPE dyspnea	5.1 ± 2.0	5.2 ± 2.7	0.93	0.1	−0.8, 0.9	1.7	−3.2, 3.4	28.4	0.76 (0.44, 0.91)
RPE leg effort	6.8 ± 2.1	7.5 ± 1.6	0.23	0.8	−0.3, 1.8	2	−3.1, 4.7	29.5	0.41 (−0.04, 0.73)
V'E/V'O_2_ at VAT	31.8 ± 4.8	32.8 ± 5.0	0.28	0.9	−0.8, 2.7	3.3	−5.6, 7.4	7.3	0.77 (0.48, 0.91)
V'E/V'CO_2_ at RCP	33.8 ± 3.7	33.8 ± 3.7	0.91	0.1	−1.2, 1.3	2.0	−3.9, 4.0	4.5	0.87 (0.60, 0.96)
V'E-vs-V'CO_2_ slope to RCP	32.2 ± 4.8	33.0 ± 5.5	0.36	0.8	−1.0, 2.6	2.8	−4.7, 6.3	6.1	0.85 (0.58,0.96)
6 MWD (*n* = 12)	502.5 ± 113.4	528.3 ± 113.1	0.010	25.8	7.5, 44.1	28.7	−30.5, 82.1	5.6	0.95 (0.65, 0.99)

*n* = 17, unless otherwise indicated

*TB1* baseline test 1, *TB2* baseline test 2, *APMHR* age-predicted maximal heart rate, *VAT* ventilatory anaerobic threshold, *RCP* respiratory compensation point, *6 MWD* 6 min-walk distance, *MD* mean difference, *SDD* standard deviation of difference, *LoA* limits of agreement, *CoV* coefficient of variation, *ICC* intraclass correlation coefficient, *CI* confidence interval, *x̄* individual average

**Table 3:** Changes in cardiopulmonary fitness during 4 weeks

	TB2	TF	MD (95 % CI) (TF-TB2)	p-value	% changes (95 % CI)
Absolute V'O_2peak_ (mL/min)	1156.4 ± 376.6	1170.0 ± 397.5	13.6 (−102.9, 130.2)	0.81	2.8 (−6.3, 11.9)
Relative V'O_2peak_ (mL/kg/min)	16.0 ± 4.9	16.2 ± 5.0	0.2 (−1.3, 1.6)	0.80	2.8 (−6.3, 11.9)
HR_peak_ (beats/min)	124.2 ± 25.2	124.5 ± 23.5	0.4 (−4.7, 5.4)	0.88	0.9 (−3.1, 5.0)
RER_peak_	1.08 ± 0.08	1.08 ± 0.10	0.001 (−0.02, 0.03)	0.89	0.2 (−2.3, 2.6)
WR_peak_ (W) (*n* = 16)	29.0 ± 13.3	30.0 ± 14.0	1.0 (−1.8, 3.8)	0.46	2.6 (−7.3, 12.6)
Absolute V'O_2VAT_ (mL/min) (*n* = 16)	637.4 ± 173.3	569.3 ± 151.3	−68.1 (−107.5, −28.7)	0.002	−9.6 (−15.1, 4.0)
Relative V'O_2VAT_ (mL/kg/min)	8.8 ± 2.3	7.9 ± 2.0	−0.9 (−1.5, −0.4)	0.002	−9.6 (−15.1, 4.0)
HR_VAT_ (beats/min)	93.8 ± 11.5	91.1 ± 7.4	−2.6 (−6.8, 1.5)	0.20	−2.1 (−6.2, 2.0)
Absolute V'O_2RCP_ (mL/min) (*n* = 11)	1018.7 ± 179.1	1055.4 ± 199.4	36.7 (−80.4, 153.7)	0.50	4.5 (−5.9, 14.9)
Relative V'O_2RCP_ (mL/kg/min)	14.2 ± 2.5	14.7 ± 2.5	0.5 (−1.0, 1.9)	0.48	4.5 (−5.9, 14.9)
HR_RCP_ (beats/min)	118.2 ± 16.8	116.8 ± 19.0	−1.3 (−8.8, 6.1)	0.69	−1.0 (−7.7, 5.7)
RPE dyspnea	5.2 ± 2.7	4.5 ± 1.9	−0.7 (−1.9, 0.5)	0.25	−0.7 (−25.2, 23.8)
RPE leg effort	7.5 ± 1.6	6.1 ± 2.2	−1.4 (−2.8, −0.2)	0.028	−16.4 (−32.4, 0.4)
V'E/V'O_2_ at VAT (*n* = 16)	32.4 ± 4.6	32.4 ± 4.2	0.1 (−1.2, 1.3)	0.93	0.5 (−3.2, 4.3)
V'E/V'CO_2_ at RCP (*n* = 11)	33.7 ± 3.8	33.5 ± 4.4	−0.1 (−1.7, 1.4)	0.86	−0.4 (−5.2, 4.5)
V'E-vs-V'CO_2_ slope to RCP (*n* = 11)	32.8 ± 5.7	32.0 ± 4.6	−0.8 (−3.4, 1.9)	0.53	−1.3 (−8.4, 5.8)
6MWD (*n* = 12)	528.3 ± 113.1	526.7 ± 114.5	−1.7 (−26.1 to 22.8)	0.89	0.1 (−5.0 to 5.2)

*n* = 17, unless otherwise indicated

*TB2* baseline test 2, *TF* follow up test, *MD* mean difference, *CI* confidence interval

### Test-retest reliability (TB1 vs TB2)

**Fig. 3:**
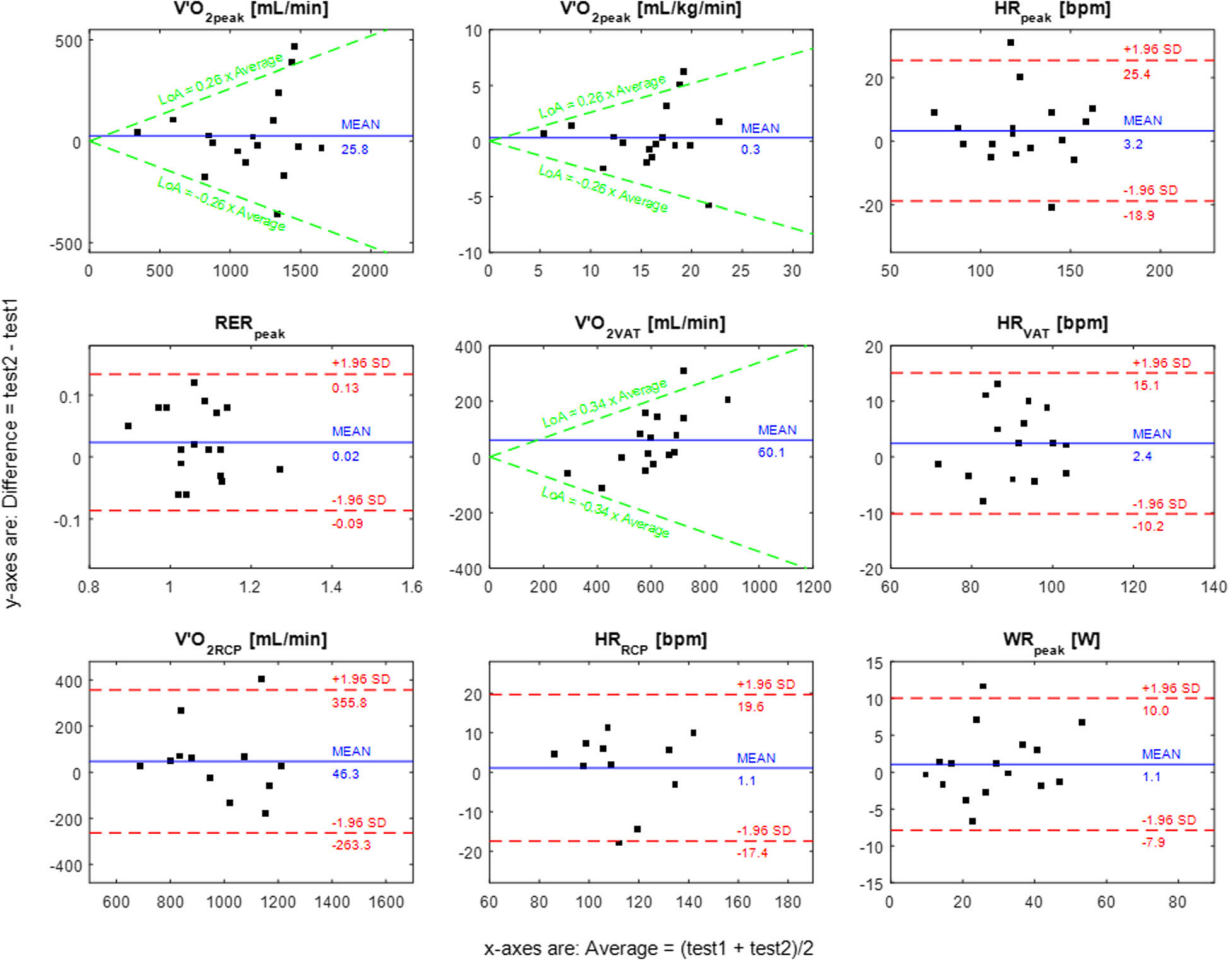
Bland-Altman plots for the main outcome measures. The differences between test 2 (TB2) and test 1 (TB1) are plotted against the average values of TB1 and TB2

The first and second baseline tests (TB1 and TB2) were 2.1 ± 2.1 days apart. The primary outcomes (V'O_2peak_, HR_peak_, RER_peak_, WR_peak_, V'O_2VAT_ and V'O_2RCP_) showed good to excellent test-retest reliability with ICCs in the range of 0.65 to 0.94 (Table 2). Overall, peak exercise performance parameters showed higher reliability compared to submaximal exercise parameters: V'O_2peak_ had a better reliability and repeatability shown by a higher ICC, a lower or equal coefficient of variation and a smaller mean difference (ICC 0.85 [95 % CI 0.64-0.94], CoV 11.6 %, MD 25.8 mL/min) compared to V'O_2VAT_ (ICC 0.67 [95%CI 0.27–0.87], CoV 14.5 %, MD 60.1 mL/min) and V'O_2RCP_ (ICC 0.65 [95%CI 0.17–0.88], CoV 11.6 %, MD 46.3 mL/min). Most cardiopulmonary exercise parameters were slightly higher in TB2: a statistically significant difference was found between the tests only for V'O_2VAT_ (575.4 mL/min in TB1 vs 635.5 mL/min in TB2, p = 0.045). For the Bland and Altman analysis, the variables V'O_2peak_, V'O_2VAT_ and HR_VAT_ as a percentage of HR reserve were found to be heteroscedastic and the limits of agreements were calculated from the log-transformed data (Table 2, Fig. 3).

Amongst the secondary outcomes, HR_VAT_, HR_RCP_, V'E/V'O_2_ at VAT, V'E/V'CO_2_ at RCP and V'E-vs-V'CO_2_ slope showed excellent reliability with ICC from 0.76 to 0.87 and CoV less than 8 %. The least reliable parameters as classified by the highest CoV (28.4 and 29.5 %) were the patients’ subjective rating of perceived exertion (RPE), both for leg effort and dyspnea.

### Changes in cardiopulmonary fitness after 4 weeks (TB2 vs TF)

The follow up test (TF) was 30.4 ± 10.2 days after the second baseline measurement (TB2). No significant changes were found in V'O_2peak_, HR_peak_, RER_peak_, WR_peak_, or V'O_2RCP_ (Table 3), but V'O_2VAT_ showed a statistically significant decrease (637.4 mL/min in TB2 vs 569.3 mL/min in TF, p = 0.002). Regarding secondary outcomes, mean RPE for leg effort in TF was lower than for TB2 but the other variables were comparable (Table 3).

## Discussion

This study had two aims: to investigate test-retest reliability and repeatability of CPET on the RATT in stroke patients with all degrees of disability; and to prospectively investigate changes in cardiopulmonary outcomes in this sample over a period of four weeks.

### Test-retest reliability

Good to excellent reliability was found in V'O_2peak_, HR_peak_, RER_peak_, WR_peak_, V'O_2VAT_ and V'O_2RCP_. Overall, there were slightly higher cardiopulmonary performance parameter values in test 2, but, amongst the formal outcome measures, only the difference in V'O_2VAT_ reached statistical significance.

The test-retest reliability of the peak cardiopulmonary exercise parameters observed here in stroke patients (V'O_2peak_ ICC of 0.85 and CoV of 11.6 %) was lower than values seen previously in healthy individuals (ICC of 0.97 and CoV of 4.1 %) exercising on the RATT [17]. The lower reliability may be explained by the higher variation in day-to-day levels of motor performance, levels of fatigue and motivation in stroke patients.

Comparing the test-retest reliability results with other devices used in stroke patients is difficult as ICCs are often reported by different ICC methods. Additionally, test-retest reliability as calculated by other measures such as CoV are scarce in the stroke literature. Results from various previous studies found that V'O_2peak_ obtained from a cycle ergometer, a semi-recumbent cycle ergometer, a treadmill and a robotics-assisted treadmill in both sub-acute and chronic stroke yielded excellent reliability with ICC ranging from 0.82 to 0.98 [10, 33–38]. However, one study in sub-acute stroke patients using a semi-recumbent cycle ergometer showed poor reliability: there was an increase of 10 % in V'O_2peak_ in the second test and an ICC for V'O_2peak_ of 0.50 [24]; the reason for this discrepancy is unclear.

Additional maximal exercise parameters showed good to excellent test-retest reliability in previous reports. The ICC of 0.90 for HR_peak_ in the present study is comparable to previous reports (0.74 to 0.97) for a cycle ergometer, a treadmill and a robotics-assisted treadmill [10, 24, 33, 35–38]. By way of comparison, and bearing in mind the differences in degrees of disability and testing devices, the ICCs for RER_peak_ and WR_peak_ of 0.78 and 0.94 found here can be contrasted with other reports in chronic stroke patients with mild to moderate disability using a cycle ergometer and a treadmill (0.72 for RER_peak_ and 0.99 for work rate) [36, 37].

For the Bland and Altman analysis, we found that V'O_2peak_, V'O_2VAT_ and HR_VAT_ as a percentage of HR reserve were heteroscedastic (Fig. 3). The finding of heteroscedasticity in V'O_2peak_ was also previously reported in stroke patients and patients with cardiac and pulmonary problems [33, 39, 40]. This means that patients with low V'O_2peak_ have lower variation in the absolute test-retest difference compared to patients with higher V'O_2peak_. This finding is important as it implies that, for future studies, it would be necessary to plan the test-retest reliability protocol for specific levels of exercise capacity (i.e. V'O_2peak_) to get the most accurate results because variations in the test-retest data are not uniform.

For submaximal exercise thresholds, the reliability of V'O_2VAT_ and V'O_2RCP_ were lower than for V'O_2peak_: ICC was 0.67 and CoV was 14.5 % for V'O_2VAT_; ICC was 0.65 and CoV was 11.6 % for V'O_2RCP_. These values, again, reflect lower reliability compared to healthy individuals on the same device: the ICC was 0.92 for both V'O_2VAT_ and V'O_2RCP_ and the CoV was 5.9 and 6.5 % for V'O_2VAT_ and V'O_2RCP_, respectively, for healthy individuals [18]. The finding of lower reliability for V'O_2VAT_ and V'O_2RCP_ compared to V'O_2peak_ was not unexpected as this was previously documented in healthy individuals, and in patients with cardiac and pulmonary problems [17, 18, 39]. This was thought to be because of day-to-day biological variability, which may be related to intrinsic factors such as daily haemodynamic fluctuations [39]. Additionally, the lower reliability may be caused by intra and inter-rater variability in the determination of the submaximal exercise thresholds. It was found that the intra or inter-rater variability was 5 to 12 % in patients with congestive heart failure and in normal subjects [41, 42].

HR_VAT_ and HR_RCP_ were shown to have excellent reliability. This suggests that the use of HR_VAT_ and HR_RCP_ to set the recommended intensity for exercise training could be implemented in practice. RPE showed the lowest reliability and repeatability. The findings in the present study support previous evidence that RPE is not an appropriate indicator of exercise intensity in stroke patients at a high-intensity exercise level [43]. V'E/V'O_2_ at VAT, V'E/V'CO_2_ at RCP, and the V'E-vs-V'CO_2_ slope showed excellent test-retest reliability with CoVs comparable to healthy individuals (CoV of 4.5 to 7.3 % in stroke patients vs CoV of 2.5 to 6 % in healthy individuals) [18].

In summary, based on this evidence, reliability and repeatability of the main CPET parameters obtained from the RATT are comparable to previous findings reported in stroke patients using standard exercise testing devices. However, the reliability and repeatability levels found here in stroke patients were generally lower than for healthy individuals on the RATT.

### Changes in cardiopulmonary fitness after 4 weeks

Apart from V'O_2VAT_, the main cardiopulmonary performance parameters did not demonstrate any statistically significant differences over the four week period. This may be because the follow up time was short and because more than half of the patients who participated in this study were in the chronic phase following stroke. The non-significant change in mean V'O_2peak_ from 16.0 to 16.2 mL/kg/min is comparable to a study in a control group of chronic stroke patients: 15.1 to 15.2 mL/kg/min in a 10-week period [37]. This reflects that without any specific exercise intervention, changes in V'O_2peak_ are unlikely to be seen over a short period.

There was a significant difference (decrease) in V'O_2VAT_ over the 4-week period (TB2 vs TF). However, based on the large and significant difference in V'O_2VAT_ between tests TB1 and TB2 (mean difference of +60.1 mL/min) and the fact that V'O_2VAT_ from TB1 was only 575.4 mL/min (Table 2), this is considered not to be clinically significant.

The observation of no changes in cardiopulmonary fitness over 4 weeks might serve as a baseline for future studies which implement a training intervention using the RATT.

### General comments

V'O_2peak_ averaged over the three tests was 16 mL/kg/min. This is in line with previous reports and shows that cardiopulmonary fitness in stroke patients is low (8 to 22 mL/kg/min, 26 to 87 % of age and sex-matched prediction) [8].

The cardiopulmonary fitness values obtained in this study need to be interpreted with caution. The V'O_2peak_ and V'O_2_ at submaximal exercise thresholds are device specific: it was found that peak and submaximal V'O_2_ were approximately 20 % lower on the RATT than the cycle ergometer in healthy individuals [17, 18]. The HR response is also device specific: the HR_peak_ of 80 % of age predicted maximal heart rate (APMHR) obtained from CPET on the RATT in this study is at the lower end of the range for HR_peak_ reported for a recumbent cycle ergometer, an upright cycle ergometer and a treadmill in chronic ambulatory stroke patients (78.2 to 94.7 % of APMHR) [34, 44, 45]. The lower values for V'O_2peak_ and HR_peak_ on the RATT compared to other devices may be because of more body support provided and a difference in the movement pattern of exercise on the RATT [17].

An RER_peak_ of > 1.0 is a recommended corroboratory criterion for detection of maximal effort in stroke patients [46]. This criterion was satisfied here in 82.4 % of all tests, even though severely disabled patients were included. This is considered a high proportion compared to previous reports of only 17.6 to 62.1 % in patients with sub-acute or chronic ambulatory stroke tested on a treadmill [44, 47, 48], 44 % of sub-acute stroke patients tested on a semi-recumbent cycle ergometer [24], and 78.9 % of sub-acute stroke patients using a cycle ergometer [49]. These differences in the proportion of patients who achieved the criterion for maximal effort points to the importance of the device employed for CPET. Patients may experience difficulty exercising on a treadmill at higher speeds because of balance problems or fear of falling and they may have problems with leg control during cycling that prevent them from reaching their maximal exercise capacity. The RATT, in contrast, provides a body harness, thigh cuffs and foot plates to secure the patients, thus enabling them to exercise to a higher intensity safely and securely.

Overall, the VAT was identified in 16/17 (94.1 %) (TB1), 17/17 (100 %) (TB2) and 16/17 (94.7 %) (TF) patients. The RCP was identified in 12/17 (70.6 %) (TB1), 14/17 (82.4 %) (TB2) and 13/17 (76.5 %) (TF) patients. In contrast, the VAT was previously demonstrated to be identifiable in only 67.3 to 83.5 % of chronic stroke patients exercising on a semi-recumbent cycle ergometer, an upright cycle ergometer or a treadmill [45]. To the best of our knowledge, there are no other studies in stroke patients that reported identification of an RCP, which is a point of substantially higher exercise intensity than the VAT, apart from our own feasibility study [15]. The finding of high proportions of patients who were able to exercise on the RATT to a sufficiently high level of intensity to allow identification of both the VAT and the RCP is regarded as an important finding. These submaximal exercise thresholds provide additional information regarding a patient’s fitness status [23, 50] and they can be used to follow up after an exercise intervention [51, 52]. Moreover, these submaximal exercise thresholds could be adopted for the prescription of individualised exercise programmes because the exercise intensity between the VAT and RCP reflects the recommended individualized moderate to high intensity exercise regime [53].

HR_VAT_ in this study was lower than the value reported in mild to moderate chronic stroke on a treadmill: here, HR_VAT_ was approximately 59 % of APMHR and in a study of Bosch et al. (*n* = 8) it was 66 % of APMHR [26]. This difference may be due to the device specific responses to exercise as mentioned above.

### Limitations

The strict patient eligibility criteria described in Methods and the exclusion of patients who had cardiac problems or who were not approved by the cardiologist for CPET led to the small sample size in this study, which may limit generalizability of the findings. Further investigation in a large cohort of stroke patients from the principal target population, that is, patients with severe disability, is warranted.

## Conclusions

These findings provide the first evidence of test-retest reliability and repeatability of the principal CPET variables using the novel RATT system and testing methodology, together with evidence of high success rates in identification of submaximal CPET threshold variables. Good to excellent test-retest reliability and repeatability were found for all submaximal and maximal CPET variables. Reliability and repeatability of the main CPET parameters in stroke patients on the RATT were comparable to previous findings in stroke patients using standard exercise testing devices. The RATT has potential to be used as an alternative exercise testing device in patients who have limitations for use of standard exercise testing devices.

## Abbreviations

6 MWD: Six-minute walk distance
APMHR: Age-predicted maximal heart rate
CoV: Coefficient of variation
CPET: Cardiopulmonary exercise testing
HR: Heart rate
HR_peak_: Peak heart rate
ICC: Intraclass correlation coefficient
LoA: Limits of agreement
MD: Mean difference
P_ET_CO_2_: Partial pressure of end-tidal carbon dioxide tension
P_ET_O_2_: Partial pressure of end-tidal oxygen tension
RATT: Robotics-assisted tilt table
RCP: Respiratory compensation point
RER_peak_: Peak respiratory exchange ratio
RPE: Rating of perceived exertion
VAT: Ventilatory anaerobic threshold
V'CO_2_: Carbon dioxide output
V'E: Minute ventilation
V'E/V'CO_2_: Ventilatory equivalent of carbon dioxide
V'E/V'O_2_: Ventilatory equivalent of oxygen
V'O_2_: Oxygen uptake
V'O_2peak_: Peak oxygen uptake
WR_peak_: Peak work rate
